# Exploring the potential of TEM analysis for understanding cooking at prehistoric feasting sites

**DOI:** 10.1038/s41598-020-70628-4

**Published:** 2020-08-12

**Authors:** Katie E. Faillace, M. George B. Foody, Richard Madgwick

**Affiliations:** 1 School of History, Archaeology, and Religion, Cardiff University, Colum Drive, Cardiff, CF10 3EU UK; 2 Archaeogenetics Research Group, Department of Biological Sciences, School of Applied Sciences, University of Huddersfield, Queensgate, Huddersfield, HD1 3DH UK

**Keywords:** Archaeology, Transmission electron microscopy

## Abstract

This study explores the utility of transmission electron microscopy (TEM) analysis of bone collagen for investigating prehistoric cooking. Approaches to cooking practices have relied principally on artefactual evidence, macroscopic bone modification, and organic residue analysis. However, direct evidence for cooking of bone has been limited. Richter and Koon successfully applied TEM analysis of collagen to determine heating to modern and medieval bones, but this method has yet to be experimentally tested using prehistoric remains. Collagen will denature at relatively low temperatures, such as during roasting, boiling, or baking. The denaturation of collagen causes predictable structural changes that can be viewed through TEM. Zooarchaeological remains of sheep and pig with minimal taphonomic modifications were analysed from four later prehistoric (c. 800–500BC) sites in Britain (n = 33). Humeri and phalanges were selected to compare elements with high and low meat yields. Samples were classified into ‘Heated’ and ‘Unheated’ groups consistent with previous studies, and variable patterns were observed between different sites and taxa. Analytical limitations have hindered the study of cooking in the past, but this study demonstrates the potential of this taphonomic method for exploring prehistoric cooking practices and provides a springboard for wider studies.

## Introduction

This research explores the potential of transmission electron microscopy (TEM) analysis for reconstructing cooking practices in prehistoric faunal assemblages, using midden sites from late prehistoric southern Britain as the testbed. Evidence for large scale feasting is common in the archaeological record of the Bronze Age–Iron Age transition in southern Britain . This is characterized by a high volume of faunal remains which accumulated rapidly, signaling the coming together of vast numbers of people and animals from widespread communities for feasts on a scale unparalleled in the British archaeological record^1–3^. These sites are commonly termed middens and provide some of the richest resources for understanding prehistoric society. They are monumental mounds of material culture, some covering 3.5 ha and comprising tens of millions of artefacts dominated by animal bone and pottery. Zooarchaeological analyses have provided insights into preparation and consumption practices through butchery marks and species and element representation^1,4–7^. However, only charring provides direct macroscopic evidence for a cooking process. This modification is typically observed on a small proportion of remains from these sites and is only likely to be present on extremities that are exposed to flames. The method of cooking often holds substantial cultural importance^8,9^, yet it remains frustratingly inaccessible through most archaeological analyses. It is particularly pertinent during this phase when the production and consumption of agricultural produce was at the very core of socio-economic change^10^.

The study of food in the past can be broken down into stages of food production: procurement, processing and cooking, consumption, and discard^11^. Food storage can also be considered between these stages. Current methodologies for inferring past foodways from osseous remains have improved markedly from an assessment of taxa, elements, and mortality profiles. Approaches include ZooMS, dental microwear, analysis of calculus and isotope analysis. Despite the range of methods for reconstructing diet, there is no certain method for identifying cooking processes in the past, except what can be extrapolated from organic residue analysis or from food remains showing evidence for charring^11^. Instead, most interpretations of archaeological cooking practices rely on theoretical and ethnographic models^12–14^. However, this invisibility of cooking in the archaeological record precludes a thorough understanding of nutritional value, labour organization, and identity^11,14^. Research by Koon^15–17^ has demonstrated the potential of TEM of bone collagen for identifying heat treatment of bones in modern experiments and archaeological remains from historic periods. This study extends this approach to later prehistoric material to explore its potential for older samples and to evaluate the practicalities of the method, while also considering the nature of cooking at these enigmatic feasting sites.

## Principles and previous application of TEM collagen analysis

Collagen, specifically Type I collagen, is the protein that makes up the majority of the organic component of bone^18,19^. It grows in bundled fibrils, analogous to rope. Prolonged or excessive heating (> 150 °C or > 9 h) will cause collagen to turn into gelatin; however, below those extremes, the process of heating causes the fibrils to denature, or, to continue the analogy, to fray^15^. The result of denaturation can be observed using TEM.

**Figure 1:**
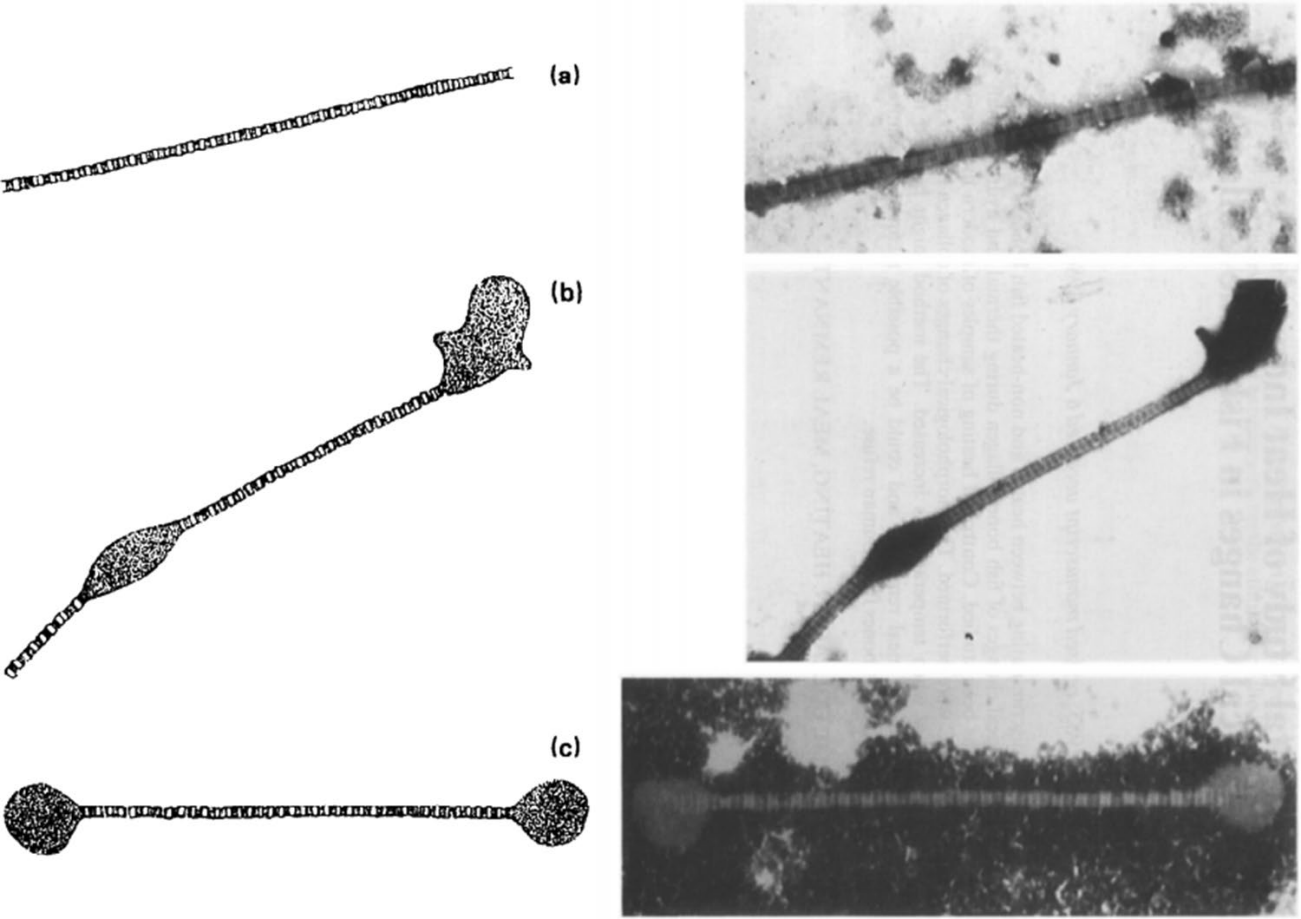
Collagen classification categories from Richter^20^: unaltered (**a**), beaded (**b**), and dumbbell (**c**). Reprinted from Journal of Archaeological Science, Vol 13, J Richter, Experimental study of heat induced morphological changes in fish bone collagen, 477–481, (1986), with permission from Elsevier.

**Figure 2:**
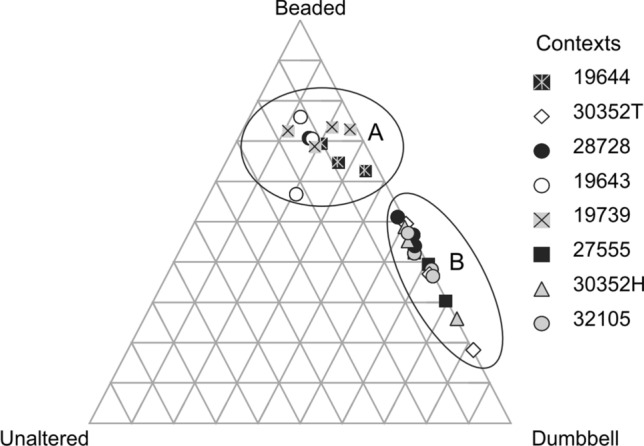
Ternary plot from Koon et al.^17^. The clusters demonstrate uncooked (**A**) and cooked (**B**) bones from Medieval York. Reprinted from Journal of Archaeological Science, Vol 37, HEC Koon, TP O’Connor, & MJ Collins, Sorting the Butchered from the Boiled, 62–69, (2010), with permission from Elsevier.

Richter^20^ first demonstrated that changes in bone collagen appearance could indicate low-temperature heating such as experienced during cooking. By experimentally heating fleshed and defleshed fish bones to 60 °C or 80 °C, or by boiling for 30 min, Richter identified three stages of collagen denaturation: Unaltered, Beaded, and Dumbbell (Fig. 1). Unaltered collagen is defined as a fibril that is long, uniform in width, and intact. Beaded collagen is identified as a fibril that is long but with areas of along the length that have expanded beyond the usual width, giving the silhouette of beads on a string. Dumbbell collagen is a fibril that is short (< 3 um), with areas of expansion at both ends, from where the formerly Beaded fibril broke at the ‘beads.’ These categories were then successfully employed to demonstrate the cooking of archaeological fish bones using a small sample (n = 7) from Neolithic Denmark^21^. Despite the initial success from Richter^20,21^, the method was not further investigated until Koon et al*.*^16^ applied this methodology by experimentally burying cooked and uncooked modern mammalian bones in a variety of soils. Similar results to Richter^20,21^ were achieved with a limited number of observed fibrils. However, they also observed denaturation of unheated collagen from acidic (pH 3.5–4.5) burial environments that would otherwise be expected in heated bones. In these instances, cooked and uncooked bones from the same acidic environment could be distinguished based on extent and quantity of denatured fibrils, establishing the importance of intra-site comparison for this method. Additionally, since taphonomic processes could cause the same alterations as seen in heating, it is possible that this method of examining collagen under TEM is chronologically or environmentally limited, as the denaturation from both heating and taphonomic processes could theoretically reach a saturation point where they are indistinguishable from each other. In a follow up study^17^, medieval cattle remains were tested from contexts where remains were likely to have been cooked (domestic waste) or not (bones far from the settlement site, ‘primary’ butchery waste). When plotted, the results of the TEM analysis naturally split into two clusters, interpreted as ‘Cooked’ and ‘Uncooked’ groups (Fig. 2). Based on this graph, each sample was classified into the category expected from the spatial zooarchaeological analysis, with the exception of one tibia that was uncooked but found among domestic waste. Koon et al.^17^ demonstrated proof of concept and archaeological application.

This method was employed alongside several other methodologies (e.g. scanning electron microscopy, X-ray spectroscopy, gas absorption) to investigate the heating of bones by Solari et al*.*^22^ and Trujillo-Mederos et al.^23^. In both cases, TEM preparation was conducted following different protocol to Koon et al.^15–17^ and analysis was undertaken on a small sample size (*n* = 2 in both studies) to confirm the results of the other methods, therefore conclusions were limited within their studies. Discerning heated bone was possible by Trujillo-Mederos et al.^23^ despite poorer image resolution due to the different preparation method. Both Solari et al*.*^22^ and Trujillo-Mederos et al*.*^23^ demonstrated the potential of TEM analysis on ancient American samples dating to 2400–1500 BC and 700–500 BC respectively. Chadefaux and Reiche^24^ used TEM alongside other methodologies (e.g. SEM–EDX, micro-PIXE/PIGE, micro-ATR-FT-IR) to investigate heat-induced modifications. Unlike the other studies, which all used the TEM to view isolated collagen, Chadefaux and Reiche investigated the mineral–organic relationship using ultrathin sections of modern and archaeological bone (n = 6). Despite the difference in preparation and the inclusion of the hydroxyapatite mineral structure, the collagen was noticeably ‘melted’ following heating at 200 °C and 220 °C; however, it is possible that the retention of the mineral structure obscured the more subtle organic changes at the lower temperature tested (150 °C). Furthermore, the experimentally heated bone in their study was heated for 1 h, so the effect of lower temperatures for longer periods of time on the mineral–organic relationship is uncertain.

Evidence for structural changes in bone collagen when exposed to heat have also been observed in studies on living animals^25^. Molecular changes in archaeological bone collagen resulting from heating have been observed when assessing the aspartic acid racemisation (AAR). A study by Bada et al.^26^ demonstrated that bones defleshed by boiling have higher D/L amino acid ratios. AAR ratios in collagen have been shown to be consistent even if the majority of collagen has been lost^27^, highlighting the effect of heat on bone collagen.

## Aims and objectives

The overarching aim of this study is to extend previous TEM research to explore its validity on prehistoric material and, if possible, to investigate the nature of cooking at these transitional feasting sites. Precise objectives are as follows:

- Investigate the utility of this method for examining cooking at prehistoric sites and whether different approaches to analysis are required to account for naturally more degraded samples of greater age.
- Assess inter-observer reliability.
- Explore patterns of cooking at later prehistoric feasting sites on this limited sample.
- Discuss the practicalities and limitations of the method’s application to archaeological material and make suggestions for future work.

## Materials and methods

An initial study was conducted on three Late Neolithic pig ulnae from the earlier (c. 2500BC) feasting site of Durrington Walls to assess if fibrils were observable on material of this age. After establishing preservation potential, the study was expanded. Pig (*Sus scrofa*) and sheep/goat (*Ovis aries/Capra hircus*) samples were selected from four Late Bronze Age to Early Iron Age sites: Whitchurch, Llanmaes, Potterne, and East Chisenbury. In terms of number of identified specimens (NISP), Whitchurch, Potterne, and East Chisenbury were predominantly sheep/goat (49%, 41% and 56%, respectively), while Llanmaes was predominantly pig (70%)^28^. From each site, five pig and five sheep/goat elements were selected, three elements of which were long bones and two elements which were ‘short’ bones (e.g. phalanges) (Table 1). Different elements were selected because it was hypothesised that short bones were less likely to be cooked, given their smaller nutritional yield meaning they are often discarded as butchery waste when optimal exploitation is not required (as would be expected at feasting sites). However, the authors acknowledge there are multiple cooking and butchery processes which could result in the heating of short bones, e.g. in a stew pot. Evidence for marrow extraction was weak with only four samples (WH06, LLM12, PTN21, and ECH33) showing partial spiraling. Macroscopic modifications were commonplace at all sites^29,30^ and are listed for the samples in Table 1. Sampling strategy targeted elements with well-preserved surface texture and no macroscopic evidence for burning or charring. Samples exhibiting weathering (WH02, LLM12, and ECH38) did not exceed Behrensmeyer stage 1^31^. Where available, % collagen yields from isotope sample preparation^3^ are given. Although most samples in this study were not subject to isotope analysis^3^, overall % collagen yields for each of the later prehistoric sites are as follows: the mean % collagen yield for Whitchurch is 11.9% (n = 26), for Llanmaes is 7% (n = 90), for Potterne is 10% (n = 60), and for East Chisenbury is 17.4% (n = 17).

**Table 1:** Details of all analysed samples.

Site	ID	Element	%Collagen yield	Taphonomy notes	Species	Date
Durrington Walls	DW1	ulna	nd	None	Pig	Late Neolithic
	DW2	ulna	nd	None		
	DW3	ulna	nd	None		
Whitchurch	WH01	humerus	nd	None	Pig	Iron Age
	WH02	humerus	nd	G, W, A		
	WH03	humerus	nd	G		
	WH04	humerus	nd	None	Sheep/goat	
	WH05	humerus	nd	A		
	WH06	humerus	nd	B, A		
	WH07	phalanx (1)	8.0	M		
	WH08	phalanx (1)	nd	G		
	WH09	phalanx (1)	nd	C	Pig	
	WH10	phalanx (1)	nd	None		
Llanmaes	LLM11	humerus	nd	None	Pig	Early Iron Age
	LLM12	humerus	nd	W		
	LLM13	humerus	nd	G		
	LLM14	phalanx (1)	nd	None		
	LLM15	phalanx (1)	nd	None		
	LLM16	humerus	nd	None	Sheep/goat	
	LLM17	humerus	nd	None		
	LLM18	humerus	nd	None		
	LLM19	phalanx (1)	nd	None		
	LLM20	phalanx (1)	nd	None		
Potterne	PTN21	humerus	nd	G, C	Pig	Late Bronze Age–Early Iron Age
	PTN22	humerus	11.4	C		
	PTN23	humerus	nd	None		
	PTN24	phalanx (1)	nd	None		
	PTN25	phalanx (1)	nd	None		
	PTN26	humerus	nd	None	Sheep/goat	
	PTN27	humerus	nd	M		
	PTN28	humerus	nd	C		
	PTN29	phalanx (1)	nd	None		
	PTN30	phalanx (1)	nd	None		
East Chisenbury	ECH31	radius	18.6	None	Pig	Late Bronze Age–Early Iron Age
	ECH32	tibia	nd	None		
	ECH33	tibia	16.5	None		
	ECH34	metapodial	9.6	M, C		
	ECH35	phalanx (3)	nd	None		
	ECH36	humerus	22.5	None	Sheep/goat	
	ECH37	humerus	nd	None		
	ECH38	humerus	20.5	W, C		
	ECH39	phalanx (1)	22.6	None		
	ECH40	phalanx (1)	25.4	None		

*G* gnawing, *B* butchery, *A* abrasion, *W* weathering, *C* calcareous concretions, *M* = mould staining.

Collagen extraction and TEM analysis methods followed the EDTA method described in Koon^15^ and are only summarised here. A transverse cross section (c. 10 mm × 10 mm) of each element was extracted using a water-cooled diamond bladed saw, then crushed with a hammer to minimize the introduction of heat. A sample of c. 60 mg was demineralised slowly over two weeks in Pyrex test tubes using 10 ml of 0.5 M EDTA (ethylenediamine tetraacetic acid), which was changed every three days. Although demineralisation is quicker using hydrochloric acid, EDTA is less likely to damage collagen^15^. After demineralisation, the samples were washed thoroughly using deionized water and left in a phosphate buffer solution (pH7) overnight.

Samples were homogenised using a laboratory homogeniser to release the collagen while simultaneously being stained in 3 ml of phosphotungstic acid (PTA). They were then centrifuged at 4 °C for fifteen minutes at 3000×*g*. The supernatant was discarded before the samples were re-suspended in 1 ml of PTA. Next, the homogenised collagen solution was pipetted onto carbon-coated Formvar grids, two per sample, which were allowed to settle for five minutes for the collagen to attach to the grid. Excess liquid was removed using filter paper by producing a wicking action, then left to dry overnight. The next day, each grid was floated on a drop of uranyl acetate dye, covered to protect the uranyl acetate from light, and left for thirty minutes as the collagen absorbed the stain. After thirty minutes, each grid was rinsed thoroughly with an ethanol and water solution, then with water alone. Excess liquid was again removed with filter paper, and the samples were left to dry overnight. At this point, the grids were ready to be analysed using the transmission electron microscope.

**Figure 3:**
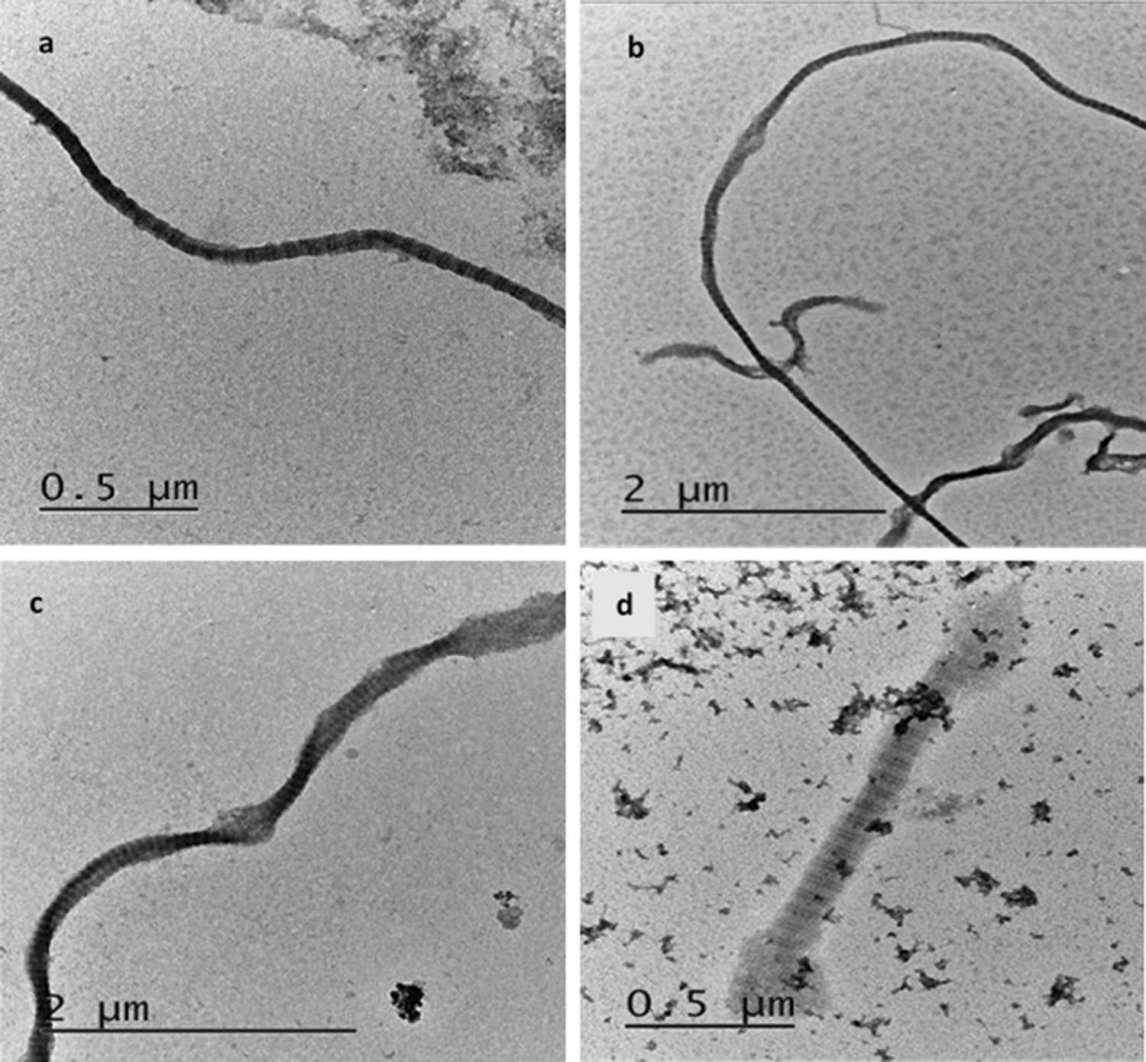
Examples of collagen classification from East Chisenbury (ECH31 and ECH36), demonstrating Unaltered (**a**), Slightly Beaded (**b**), Beaded (**c**), and Dumbbell (**d**) fibrils.

Transmission electron microscopy (TEM) was performed on a JEOL JEM-2100 operating at 200 kV, and images were taken of all identified collagen fibrils. Quantification differed from Koon^15^. One grid per sample was extensively imaged, with each visible fibril being quantified. Collagen was then classified as Unaltered, Beaded, or Dumbbell from the images (Fig. 3). A fourth category of ‘Slightly Beaded’ was recorded for methodological consideration, and areas of material that were most probably amorphous collagen were noted during recording but not included in the analysis. Samples with fewer than 30 discrete identifiable collagen fibrils were excluded from further analysis. This figure was chosen as a minimum for statistical reliability with the assumption that the collagen that was successfully suspended on the grids was a truly random (representative) sample of the collagen available from the bone sample. This assumption is implicit in the method.

## Results and discussion

### Preservation

Discrete, identifiable collagen was present in all but two samples, where only amorphous collagen that could not be reliably examined remained. However, a total of ten samples had to be excluded from further analysis, as fewer than 30 collagen fibrils were observable (Table 2), leaving 33 samples. Preservation varied by site. Llanmaes samples were most degraded, with five failing to meet the threshold. Durrington Walls was also greatly affected, with two out of three samples having too few observable fibrils.

**Table 2:** Classification of discrete collagen fibrils by sample.

ID	Unaltered	Beaded	Dumbbell	Amorphous	Total
DW1	3	15	22	5	40
DW2	0	0	4	5	**4**
DW3	0	1	5	3	**6**
WH01	5	64	3	0	72
WH02	1	45	4	1	50
WH03	1	13	18	3	32
WH04	0	31	37	6	68
WH05	0	25	17	0	42
WH06	0	0	0	0	**0**
WH07	0	16	19	1	35
WH08	1	27	6	0	34
WH09	0	12	25	1	37
WH10	0	4	0	0	**4**
LLM11	1	10	4	2	**15**
LLM12	2	15	23	4	40
LLM13	0	0	0	3	**0**
LLM14	0	10	0	0	**10**
LLM15	8	20	2	2	30
LLM16	0	11	3	1	**14**
LLM17	0	2	2	4	**4**
LLM18	4	44	10	2	58
LLM19	3	31	7	0	41
LLM20	3	40	3	0	46
PTN21	3	53	21	4	77
PTN22	0	41	14	1	55
PTN23	6	35	3	2	44
PTN24	0	0	0	3	**0**
PTN25	0	14	28	7	42
PTN26	7	29	3	0	39
PTN27	0	1	1	0	**2**
PTN28	0	25	47	4	72
PTN29	7	37	1	0	45
PTN30	0	41	5	0	46
ECH31	0	20	32	3	52
ECH32	2	25	21	3	48
ECH33	2	40	29	0	71
ECH34	1	30	12	0	43
ECH35	3	34	16	3	53
ECH36	9	57	16	1	82
ECH37	3	30	27	0	60
ECH38	15	28	3	0	46
ECH39	4	47	19	0	70
ECH40	13	46	12	2	71

Bolded numbers highlight samples with fewer than 30 discrete fibrils.

### Inter-observer error

Inter-observer error was assessed between a practised observer (KEF) and a novice observer (MGBF). Agreement was measured using Spearman’s rho correlation (Table 3) and significance was set at *p* < 0.01. Spearman’s rho correlation was used because it is nonparametric and applies to ordinal data, and was calculated using IBM SPSS Statistics 25. Agreement between observers was significant in all classification categories. Correlation was strong for the Dumbbell category, but moderate for the other two categories. This could be influenced by the fact that the Dumbbell category is the only one with a quantitative measurement in its definition, where Dumbbell fibrils are less than 3 µm, making them empirically easier to identify. Observers reviewed the classifications together and found that the main reason for discrepancies in Unaltered fibrils was the visualising method. Quantifying collagen from still images rather than within the TEM resulted in Unaltered collagen fibrils being counted multiple times by the second observer, as it was sometimes difficult to identify overlapping regions across multiple images. Therefore, observer experience is thought to be the main reason for the overall discrepancies. Despite these differences, quantified results between observers were significantly correlated; therefore, method reliability was considered sufficient to continue with analysis.

**Table 3:** Spearman's rho correlation for inter-observer agreement.

	N	Correlation	*p* value
Unaltered	32	0.527	< 0.001
Beaded	32	0.585	0.001
Dumbbell	32	0.803	< 0.001

All samples included in analysis were included for inter-observer agreement testing, except for the sample from Durrington Walls (DW1). Significance set at *p* < 0.01.

### Sites

Three samples from Late Neolithic Durrington Walls were analysed first to assess the preservation of collagen structures in prehistoric material of an earlier date. Durrington Walls was chosen for the preliminary assessment due to prior success extracting collagen for isotope analysis^32^. Presence of collagen was therefore expected but it was unclear whether the fibril structures would be identifiable to the categories used for TEM analysis. Though Richter^21^ had success with Neolithic fish remains, the sample size was small and collagen survival would not be expected to be the same in mammalian remains. Sample sizes in Solari et al*.*^22^ and Trujillo-Mederos et al.^23^ were also small. Remains from Durrington Walls revealed discrete collagen fibrils in all samples, including a range of Unaltered, Beaded, and Dumbbell fibrils in sample DW1 (Table 2). Despite preserved collagen fibrils in all samples, only one from Durrington Walls achieved the threshold of 30 observable fibrils. Nonetheless this demonstrates the potential of the approach for assessing the cooking of prehistoric mammalian remains. The more substantial samples from middens sites allow tentative statements to be made surrounding patterns of cooking.

### Whitchurch

**Figure 4:**
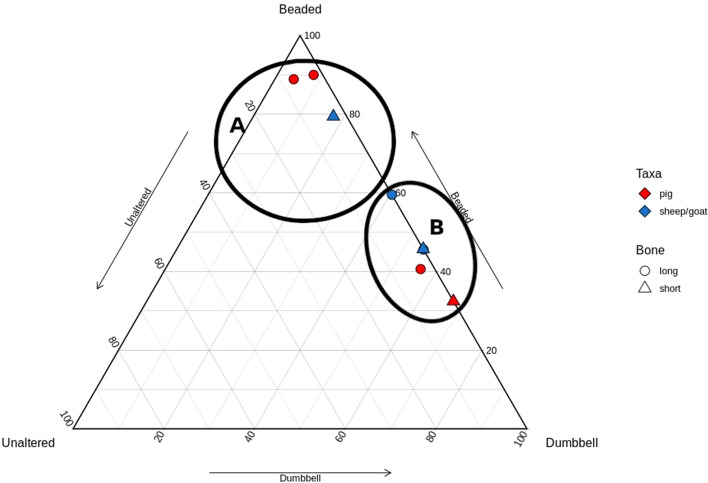
Ternary plot of Whitchurch samples; two natural groupings are demarcated.

When plotted, the samples from Whitchurch formed two groups (Fig. 4). Pig and sheep/goat were represented in both groups, as were long and short bones. The breakpoint occurred along the Beaded-Dumbbell axis, with one group clustering near the Beaded point (indicating mostly Beaded collagen with some other types), and one group clustering along the Beaded-Dumbbell axis (indicating few/no Unaltered fibrils). These clusters mimic the plot of Koon’s^17^ ‘Uncooked’ (A) and ‘Cooked’ (B) groups, respectively. No pattern was identifiable by species or element, as both element and species were split between groups.

### Llanmaes

**Figure 5:**
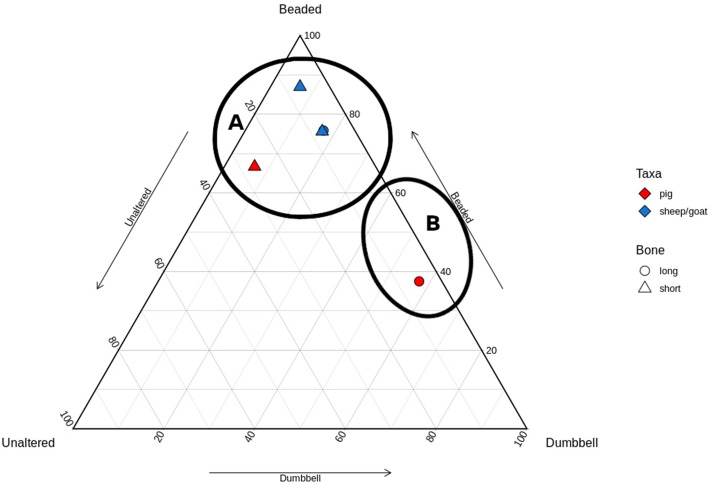
Ternary plot of Llanmaes samples; two natural groups are demarcated.

The samples from Llanmaes were much reduced by poor preservation. When plotted, there was greater distance between points, but two groups emerge in broadly the same location as the Whitchurch sample (Fig. 5). The group along the Beaded-Dumbbell axis was represented by a single pig long bone, while the group near the Beaded point was mostly sheep/goat mixed elements and one pig phalanx. Unfortunately, the reduction in this site’s sample size makes interpreting the pattern difficult, particularly when four of the five excluded elements were long bones making the remaining sample skewed towards short bones.

### Potterne

**Figure 6:**
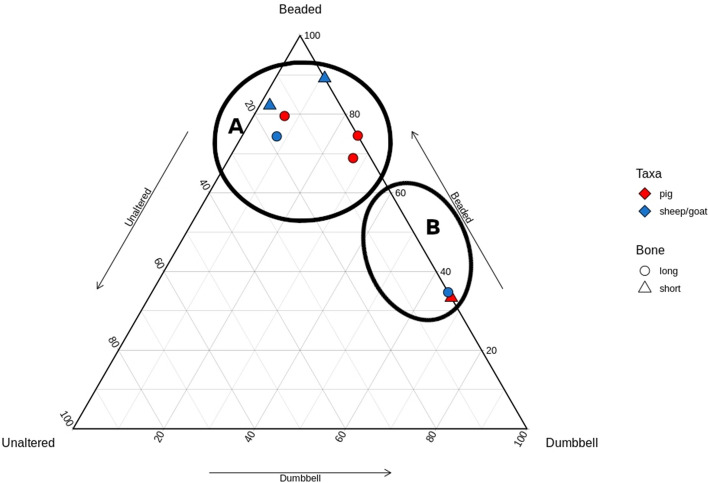
Ternary plot of Potterne samples; two natural groups are demarcated.

The samples from Potterne were unevenly split but followed the same natural separation. The cluster near the Beaded point comprised all the pig long bones and a mix of sheep/goat elements. The cluster along the Beaded-Dumbbell axis included one sheep long bone and one pig short bone (Fig. 6).

### East chisenbury

**Figure 7:**
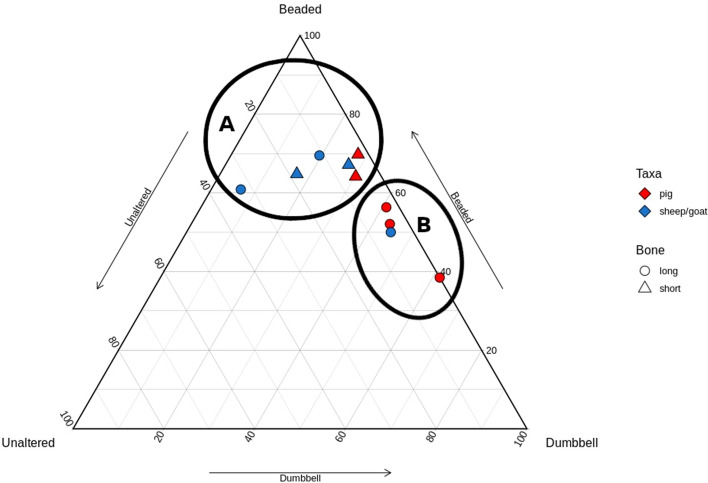
Ternary plot of East Chisenbury samples; two natural groups are demarcated.

The samples from East Chisenbury were the best preserved, with all bones included in analysis. These samples also exhibited the greatest variation in plot location (Fig. 7). Though two groups were discernable along the same axes, their spatial separation was more limited. The cluster along the Beaded-Dumbbell axis was exclusively long bones, mostly pig, and the cluster near the Beaded point was mostly sheep/goat with a mix of elements.

### Site trends

**Figure 8:**
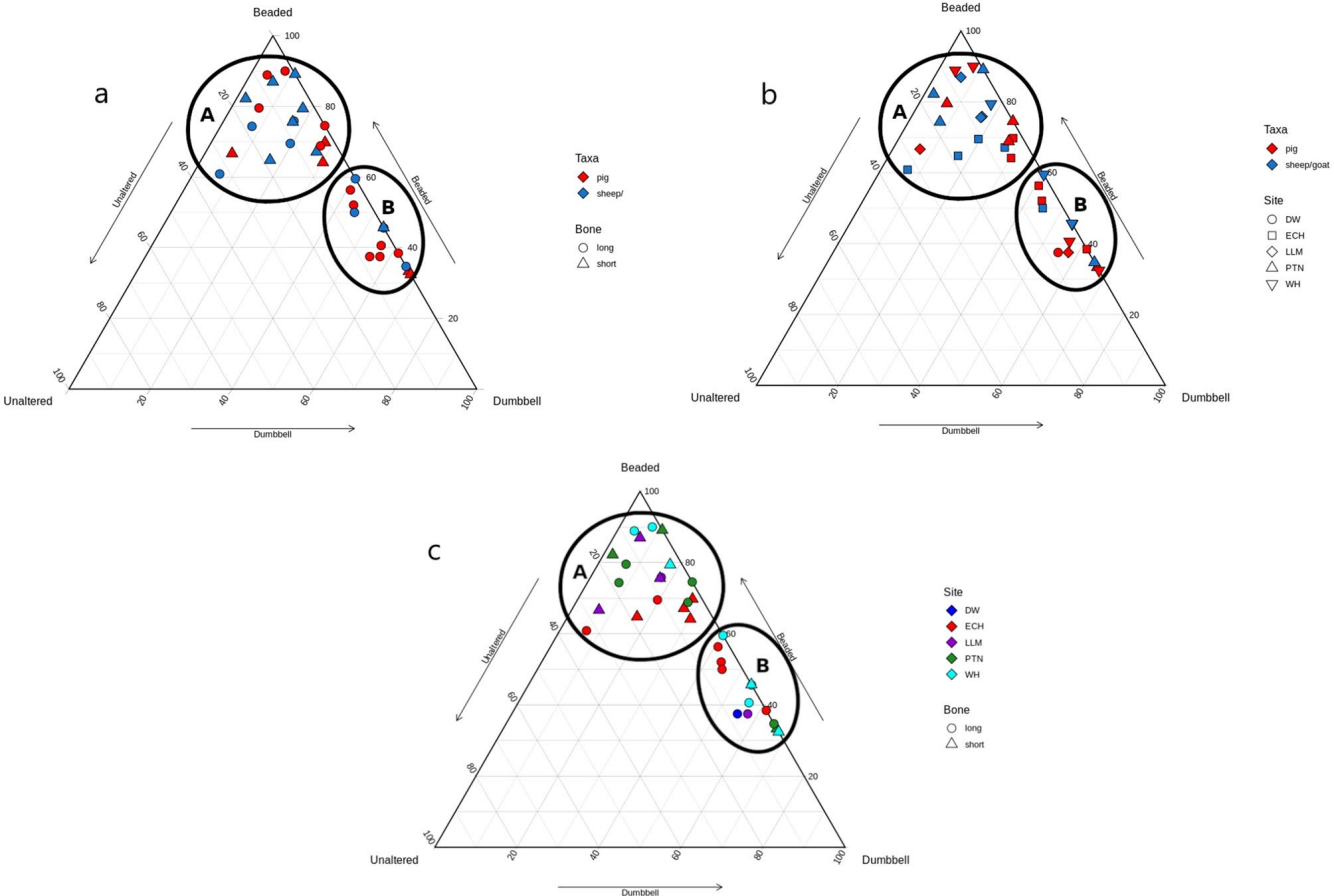
Ternary plot of all analysed samples. (**a**) presents the samples by element and species, (**b**) presents the samples by species and site, and (**c**) presents the samples by element and site. Cluster A represents Unheated samples and cluster B represents Heated samples. Categorisation is consistent with that of Koon^17^.

In Figure 8a–c all samples have been plotted together, and classification of samples into two groups was consistent with their groupings in intra-site analysis. Viewing the samples together resulted in the same natural break points as seen in the site-specific graphs. Again, the overall pattern is consistent with Koon’s^17^ plot in regards to cluster regions and shape, with a cluster along the Beaded-Dumbbell axis which is close to the axis indicating few to no Unaltered fibrils, and a cluster nearer the ‘Beaded’ point which has greater spatial separation from the axes, indicating greater proportions of Unaltered fibrils. Quantitatively, the breakpoint in this study and Koon’s^17^ study occurs along the Beaded-Dumbbell axis at the point representing samples greater or less than 40% Dumbbell. Given the consistency in natural breakpoints between sites and compared to Koon’s study, we can reasonably conclude that these groups are ‘Heated’ and ‘Unheated’ respectively.

Figure 8a presents the samples by element and species, while Fig. 8b and c compare species and element to site, respectively. Across all sites, long bones dominate the Heated group showing that these elements were more likely to be cooked than short bones. This is unsurprising given the difference in calorific yield from meat, grease, and marrow between element classes. It is perhaps unsurprising that food preparation at these feasting sites, which surely saw conspicuous consumption involving a great deal of surplus, less frequently involved the stewing of low-yield extremities. However, there is a greater diversity of elements in the Unheated group, with almost half of the long bones analysed within the Unheated group. This is true for both species, meaning that for both pigs and sheep/goats, long bones were equally likely to be cooked or uncooked. Pig short bones are slightly more common than sheep/goat in the Heated group, while nearly all sheep/goat short bones are in the Unheated group. Sheep/goat are therefore less likely to be heated (cooked) in articulation (or less likely to have short bones added to a stew pot) than pig, despite their greater representation on most sites. This may indicate socially circumscribed practices relating to the processing of different taxa (perhaps relating to greater grease/fat yields in pigs), something that is evidenced in the butchery patterns at Llanmaes^1^, although basing interpretation on such small samples is ill-advised. Despite long bones being processed in similar ways, with a near equal likelihood of cooked or not cooked, the variation in short bone representation in each group indicates greater processing of sheep/goat prior to cooking (Table 4). While this could be influenced by processing for non-food products (e.g. skins), given the specialized focus on feasting at these sites, the difference is more likely to relate to preferential consumption.

**Table 4:** Number of samples classified as heated or unheated according to element type.

	Long		Short	
	Heated	Unheated	Heated	Unheated
Pig	6	5	2	3
Sheep/Goat	4	4	1	7
Grand total	10	9	3	10

### Additional ‘slightly beaded’ category

During initial research on samples from Durrington Walls, it was apparent that there was a wide range of appearances for Beaded fibrils, with some fibrils having small, discrete expansions along an otherwise tightly structured fibril, and others having numerous, large expansions along a broadly structured fibril (Fig. 3).

**Figure 9:**
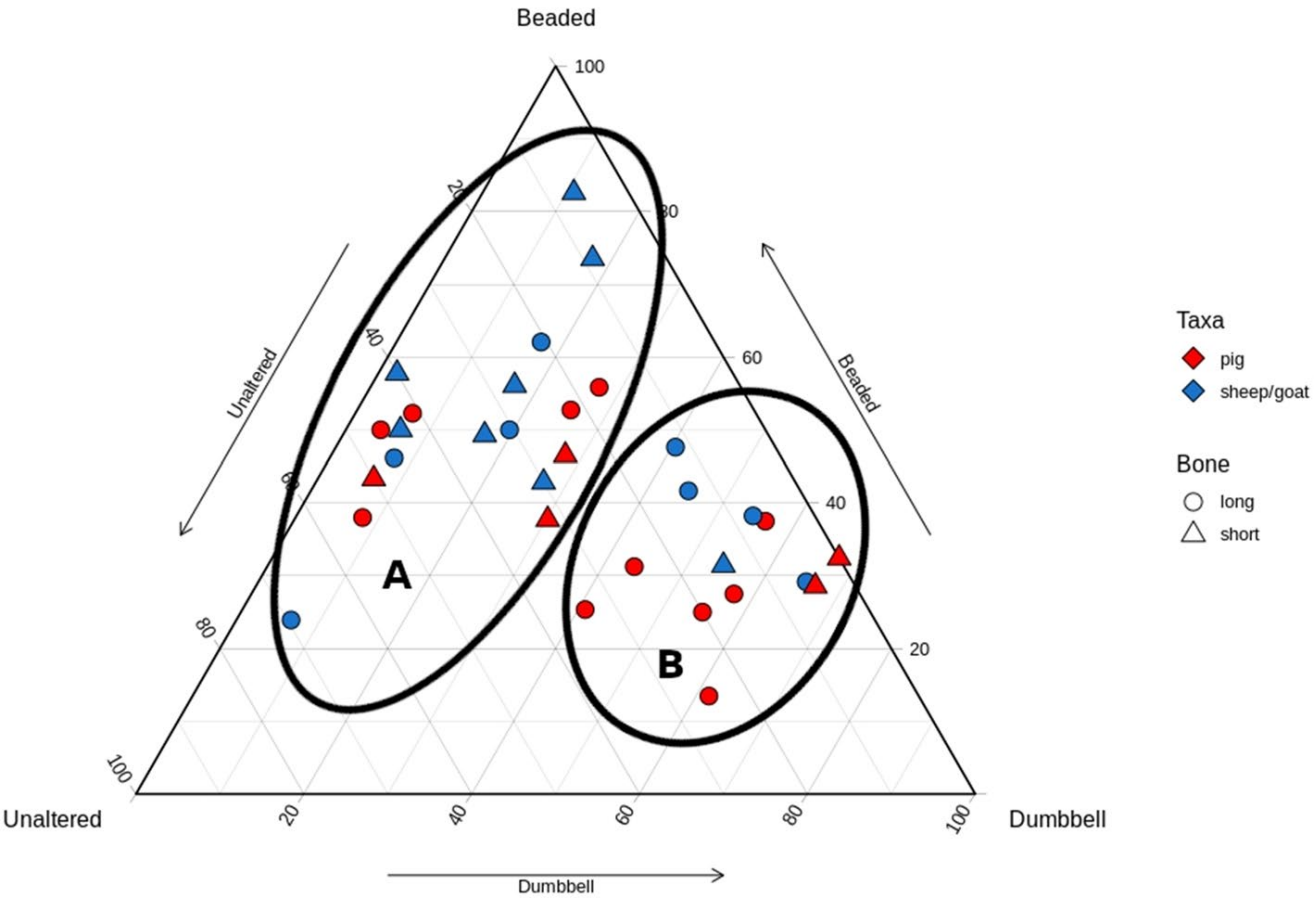
Ternary plot of all samples, where Slightly Beaded fibrils were included with Unaltered under the assumption that Slightly Beaded changes were the result of burial environment, not heat treatment. The two groups are consistent with the samples’ classification from the earlier analysis, and demonstrate that distinction is still possible, though less obviously two distinguished groups.

With consideration to Koon’s^16^ results demonstrating subterranean taphonomic influence on collagen degradation, it was hypothesized that fibrils that were Slightly Beaded (i.e. mostly Unaltered in appearance except for a few small areas of expansion) would be more representative of taphonomic degradation, while more Beaded fibrils would be representative of heating changes before burial. Therefore, during the wider analysis, observers classified Beaded collagen as either “classically” Beaded or Slightly Beaded to test this hypothesis (see Fig. 3). The data was analysed and compared to the results above. To compare on the same scale, the Slightly Beaded fibrils were subsumed under the Unaltered category and plotted as such (Fig. 9). Although the graph indicates clustering of the Cooked and Uncooked Groups, the plots within each group are more spread out and the separation between the two clusters is not as obvious. Though reassuring that the results are consistent, the difficulty in distinguishing the clusters in this format suggests the need for additional study to understand the complexity of distinguishing pre- and post-depositional denaturation. Targeted sampling could specifically investigate sites with differing geologies. Inter-observer error was calculated for these categories using Spearman’s rho correlation. Agreement for the Slightly Beaded classification was moderate and significant, but agreement for “classically” Beaded was low and insignificant (Table 5). The poor agreement between observers would suggest the definitions need to be revised in future studies investigating the difference between subterranean taphonomic denaturation and thermal denaturation.

**Table 5:** Spearman's rho correlation for inter-observer agreement of the additional 'Slightly Beaded' and "Classically" Beaded' categories.

	N	Correlation	*p* value
Slightly beaded	32	0.639	< 0.001
“Classically” beaded	32	0.176	0.335

All analysed samples were included except DW1. Significance was set at *p* < 0.01.

## Methodological considerations

One objective of this study was to examine the practicalities of employing the method and assess ways that might enhance future uptake. The potential of the technique is clear, and it remains one of the only direct methods for reconstructing cooking practices. However, its application has been very limited with only a handful of studies published. The temporal and monetary investment of investigation is likely to be one factor, as this means large sample sets are difficult to achieve. However, this has not prevented the wide-spread uptake of other high-resolution analyses of faunal remains (e.g. sequential δ^18^O isotope analysis of enamel). Sample preparation is relatively laborious, but this has also not precluded the wider uptake of other molecular analyses.

The greatest cost incurred in the application of TEM analysis of collagen is the operation of the TEM itself. It is exceptionally rare for archaeology departments to have sole use of a TEM and therefore full training and long-term use (often with an hourly charge) can be challenging to achieve. Ideally, grids could be scrutinised at length during live TEM analysis. However, the potential of a more streamlined approach was tested in this study. Grids were scanned by a professional TEM operator and micrographs taken for review. Quantifying fibrils could then be undertaken slowly and systematically. Classifications could easily be revisited and it was possible to conduct an independent inter-observer error analysis.

In comparison to Koon’s^15–17^ results, the grids in this study contained fewer collagen fibrils overall, highlighting a drawback of quantification from micrographs rather than live TEM analysis. As quantification was not undertaken during live TEM analysis, the number of observable fibrils was unknown at the point micrograph retrieval, but every effort was made to provide a representative range of micrographs. Consistency between spatial patterning on ternary plots from this study and previous studies^17^ demonstrates that despite fewer fibrils present, similar patterns emerge. This study suggests that, as long as the assumption of random sampling is not violated (e.g. recording all available fibrils, or up to 100), a smaller fibril sample size is informative. To test this assumption with the available material, samples with fibril counts over or equal to 50 were revisited (*n* = 14). Four random sub-samples of approximately 30 fibrils from each sample were compared to the total count, as well as sub-samples of 50 and 70 where possible (see Supplementary Material for more details). Multiple pairwise chi-square comparisons with Bonferroni correction were conducted between observed groups. Only one sample (ECH31) showed significant differences between groups at Bonferroni corrected level, though the ‘Cooked’ classification remained unchanged across groups. In all except one sample (ECH33), classification as cooked or uncooked was consistent with the initial analysis for each group. These are promising preliminary results to support the assumption that 30 fibrils can be representative of the overall sample, but it is recommended that future work investigates this assumption more systematically, such as by conducting a bootstrap resampling analysis on larger datasets of 30, 50, 70, and 100 fibrils and beyond. This approach to analysis requires further testing to demonstrate its validity and reliability. However, it has the potential to widen uptake and thus enhance the understanding of cooking practices in wide-ranging contexts.

Although observer agreement was acceptable, there was stronger correlation between observers for the quantifiable Dumbbell category. Reliability could therefore be improved by refining the Unaltered and Beaded categories to have objective definitions or be classified by objective technology (e.g. machine learning elliptical Fourier analysis or geometric morphometrics), but larger reference samples are needed.

Finally, the interaction between the organic and mineral components could be explored further in future studies. A comprehensive, experimental study comparing ultrathin bone sections as described by Chadefaux and Reiche^24^ and extracted collagen from the same samples would improve our understanding of the microscopic processes of cooking. A study by Bada et al.^26^ analysed the effects of amino acid racemisation upon archaeological samples. A further potentially productive future direction would be to explore the use of the discarded supernatant from the process to examine circular dichronism spectra as a possible further proxy for archaeological cooking.

## Conclusions

This study demonstrates the potential for TEM analysis to enhance understanding of prehistoric cooking practices. The main aim of the study was to assess the application of the method on prehistoric material and positive results have been produced. Tentative distinctions between heated and unheated bone were possible at all Bronze Age and Iron Age sites, though this sample size does not fully represent the diversity of prehistoric sites. This sample represents a tiny fraction of the faunal assemblages from each site and therefore it has little interpretative potential for reconstructing cooking and feasting practices on a site- or period-wide scale. However, it demonstrates the efficacy of the approach and the potential of wider study. Preliminary indications from the data suggest that meat from pigs was more often cooked in articulation than meat from sheep/goats and that the practice of cooking sheep/goat meat potentially included more processing (e.g. filleting) prior to heat exposure. The presence of short bones with evidence for cooking suggest complete limbs or whole animals were sometimes cooked at once, as one might expect from a spit-roast, but with so few short bones within the Heated group, it is unlikely to be the most common method for cooking. These bones appear to have frequently been discarded with no cooking, suggesting (as expected) that optimal exploitation of calories from the carcass was not required at these feasting sites.

At present, this research represents the largest TEM study conducted on prehistoric material. It has extended the understanding of TEM analysis of collagen from archaeological bone and identified further avenues for enquiry. Application of this method to larger sample sizes from these feasting sites is needed to make site-specific conclusions and would allow further comparisons between regions or over time to be made. Improved understanding of the representativeness of each grid of collagen is needed, and further studies using this method should also engage with the evidence from organic residues in pottery to identify practices relating to food preparation in different vessel types. Machine learning also has the potential to enhance the efficacy of fibril quantification and may prove a profitable avenue for future research.

Archaeologists have moved on from the processualist perspective that observes food and diet as merely a biological necessity; cooking too must be viewed through a cultural lens. This method provides an alternative approach to understanding cooking activities in the past.

## Supplementary information

Supplementary information.

## Data Availability

All data needed to evaluate the conclusion in this paper are present in the paper and its Supplementary Information file. Additional data generated and/or analysed during this study are available from the corresponding author on request.
