# Supplementary material for: Exploring the potential of TEM analysis for understanding cooking at prehistoric feasting sites
Source: Sci Rep. 2020 Aug 12;10:13635. doi: 10.1038/s41598-020-70628-4 (PMC7423987; doi:10.1038/s41598-020-70628-4)
Supplement: Supplementary file 1 — Supplementary information. [file 41598_2020_70628_MOESM1_ESM.docx]

**Exploring the potential of TEM analysis for understanding cooking at prehistoric feasting sites – Supplementary Information**

Katie E Faillace^1^*, M George B Foody^2^, Richard Madgwick^1^

^1^School of History, Archaeology, and Religion, Cardiff University, Colum Drive, Cardiff, CF10 3EU
^2^Archaeogenetics Research Group, Department of Biological Sciences, School of Applied Sciences, University of Huddersfield, Queensgate, Huddersfield, HD1 3DH

*corresponding author: [FaillaceKE@cardiff.ac.uk](mailto:FaillaceKE@cardiff.ac.uk)

To assess whether 30 fibrils is a valid minimum to represent patterns of degradation in a sample, samples with at least 50 recorded fibrils were re-assessed (n=14). Fibril category counts had been meticulously recorded per image. This enabled us to randomize the order of the counts by randomizing the images. Four different sub-samples of approximately 30 fibrils were created; this total is approximate because images were taken in their entirety, so if 10 images had a total of 29 fibrils, and 11 images had a total of 34 images, only the counts from the first 10 images were taken for analysis.

The sub-samples were created by taking the first 30 fibrils in the order they were originally analysed, the last 30 fibrils, and then the first 30 fibrils from two randomised image orders. Where fibril counts were great enough, the first 50 and first 70 fibrils were also grouped from the original recorded order.

Counts, percentages and ternary plots for each re-assessed subsample are provided below. As seen in the plots, each grouping is broadly consistent with the original analysis, with the exception of ECH33. However, the original analysis of ECH33 is on the border of the proposed distinction between ‘Cooked’ and ‘Uncooked’ (40% or more of fibrils classified as dumbbell are ‘Cooked’) and therefore interpretation of this samples was, in any case, ambiguous.

Multiple pairwise chi-square comparisons were used to identify significant differences in the classification of dumbbell fibrils (that most are informative for investigating cooking) between groups for each sample. Significance values are presented in tables for each sample below. Multiple pairwise testing such as this involves repeat testing of different configurations of the same dataset. This means tests are not entirely independent of each other and therefore there is a greater chance of type 1 error, the erroneous rejection of the null hypothesis. A common approach for addressing this issue is to employ a Bonferroni corrected significance level (Rice 1989). This more stringent significance value involves dividing the standard p value of 0.05 by the number of multiple pairwise comparisons. Therefore, depending on the number of defined groups significance levels of 0.005 (for samples with 10 pairwise tests), 0.0033 (15 pairwise tests) and 0.0023 (21 pairwise tests) were employed. In the results tables below, differences that are significant at the Bonferroni corrected level are shaded yellow and those that are only significant at the standard 0.05 level (and should therefore not be considered a truly significant difference) are shaded grey.

The pairwise chi-square comparisons support the qualitative conclusions from the tern plots. Of the 196 pairwise tests only two (both on ECH31) produced a significant difference at the Bonferroni corrected level, and this could be expected from the spread of points on the graph (Figure S8(b)), however, this spread does not affect its classification as ‘Cooked.’ A further 11 tests were significant at the standard 0.05 level, including two from ECH33, whose classification changed across subsamples. Ten of the 14 samples produced no significant differences at either level. Although basing interpretations on larger numbers of fibrils is desirable the very rare significant differences between the subsamples demonstrates that analysing 30 fibrils will generally provide comparable results to larger samples (i.e. 70+). Therefore, degraded samples with fewer observable fibrils are still useful for assessing cooking practices.

**Reference**

Rice, W.R. Analyzing tables of statistical tests. *Evolution* **43**, 223-225. (1989)

Below are the counts and percentages per observation group, the ternary plots of the observation groups, and the pairwise chi-square comparisons of Dumbbell fibrils between observation groups per sample.

| **WH01** | Unaltered **n**/% | Beaded **n**/% | Dumbbell **n**/% | Total n |
| --- | --- | --- | --- | --- |
| First 30 | **2**/6.5% | **29**/93.5% | **0**/0% | **31** |
| Last 30 | **3**/10% | **24**/82.8% | **2**/6.9% | **29** |
| Random 30 A | **2**/6.7% | **28**/93.3% | **0**/0% | **30** |
| Random 30 B | **2**/6.9% | **26**/89.7% | **1**/3.4% | **29** |
| First 50 | **2**/4.1% | **45**/91.8% | **2**/4.1% | **49** |
| Original Counts (all) | **5**/6.9% | **64**/88.9% | **3**/4.1% | **72** |

Supplementary Table S1(a). Counts and percentages of classified fibrils by sub-sample of WH01.


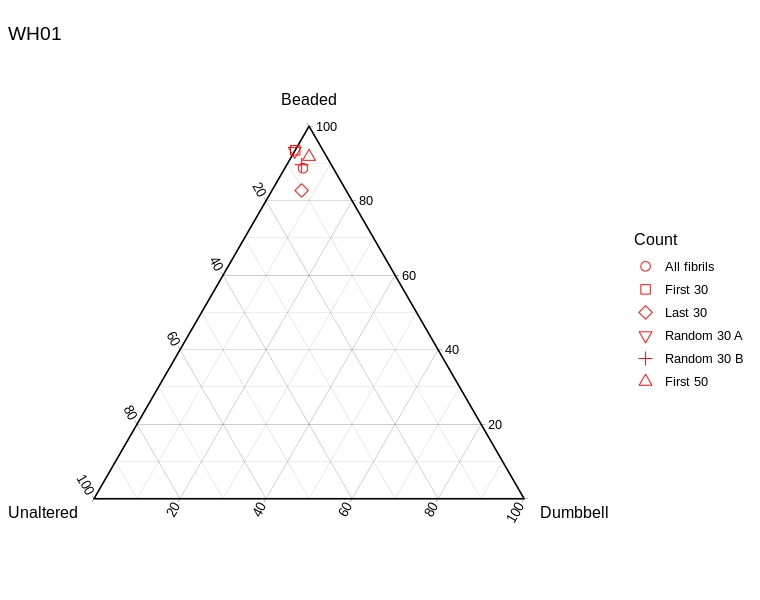


Supplementary Figure S1(b). Ternary plot representing the variation of re-assessed sub-samples of WH01.

| **WH01** (n=72) | First 30 | Last 30 | First 50 | Random 30 A | Random 30 B |
| --- | --- | --- | --- | --- | --- |
| First 30 | x |  |  |  |  |
| Last 30 | 0.137 | x |  |  |  |
| First 50 | 0.255 | 0.585 | x |  |  |
| Random 30 A | NA | 0.143 | 0.262 | x |  |
| Random 30 B | 0.297 | 0.553 | 0.888 | 0.305 | x |
| All | 0.249 | 0.567 | 0.982 | 0.256 | 0.867 |

Supplementary Table S1(c). Multiple pairwise chi-square comparisons for the representation of dumbbell fibrils in different sub-samples for WH01.

| **WH02** | Unaltered **n**/% | Beaded **n**/% | Dumbbell **n**/% | Total n |
| --- | --- | --- | --- | --- |
| First 30 | **0**/0% | **28**/93.3% | **2**/6.7 | **30** |
| Last 30 | **1**/3.5% | **26**/89.7% | **2**/6.9% | **29** |
| Random 30 A | **1**/3.1% | **29**/90.6% | **2**/6.3% | **32** |
| Random 30 B | **0**/0% | **27**/87.1% | **4**/12.9% | **31** |
| Original Counts (all) | **1**/2.0% | **45**/90.0% | **4**/8.0% | **50** |

Supplementary Table S2(a). Counts and percentages of classified fibrils by sub-sample of WH02.


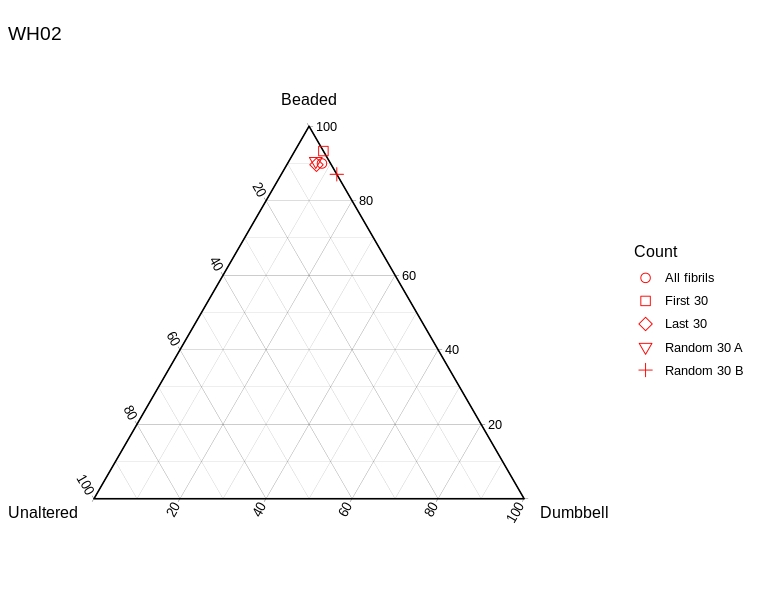


Supplementary Figure S2(b). Ternary plot representing the variation of re-assessed sub-samples of WH02.

| **WH02** (n=50) | First 30 | Last 30 | Random 30 A | Random 30 B |
| --- | --- | --- | --- | --- |
| First 30 | x |  |  |  |
| Last 30 | 0.972 | x |  |  |
| Random 30 A | 0.947 | 0.919 | x |  |
| Random 30 B | 0.698 | 0.438 | 0.638 | x |
| All | 0.826 | 0.858 | 0.767 | 0.737 |

Supplementary Table S2(c). Multiple pairwise chi-square comparisons for the representation of dumbbell fibrils in different sub-samples for WH02.

| **WH04** | Unaltered **n**/% | Beaded **n**/% | Dumbbell **n**/% | Total n |
| --- | --- | --- | --- | --- |
| First 30 | **0**/0% | **8**/26.7% | **22**/73.3% | **30** |
| Last 30 | **0**/0% | **18**/58.1% | **13**/41.9% | **31** |
| Random 30 A | **0**/0% | **15**/50.0% | **15**/50.0% | **30** |
| Random 30 B | **0**/0% | **11**/33.3% | **22**/66.7% | **33** |
| First 50 | **0**/0% | **27**/50.0% | **27**/50.0% | **54** |
| Original Counts (all) | **0**/0% | **31**/45.6% | **37**/54.4% | **68** |

Supplementary Table S3(a). Counts and percentages of classified fibrils by sub-sample of WH04.


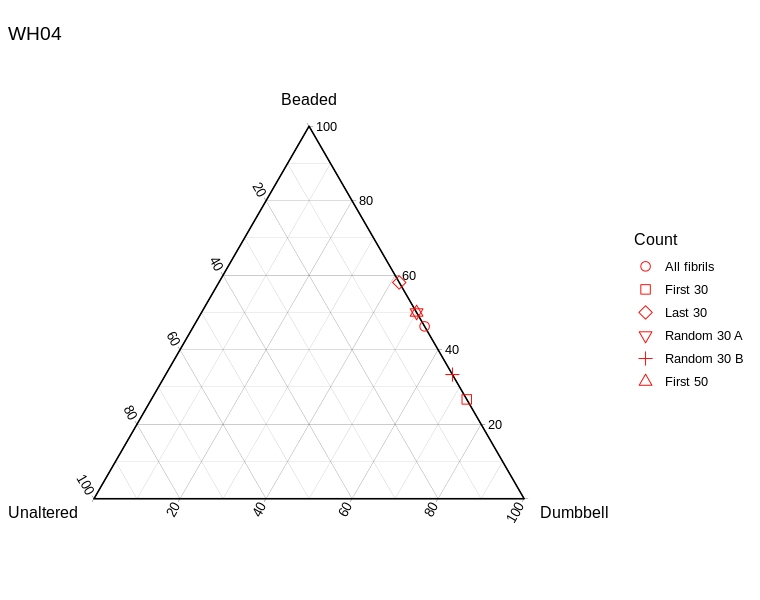


Supplementary Figure S3(b). Ternary plot representing the variation of re-assessed sub-samples of WH04.

| **WH04** (n=68) | First 30 | Last 30 | First 50 | Random 30 A | Random 30 B |
| --- | --- | --- | --- | --- | --- |
| First 30 | x |  |  |  |  |
| Last 30 | 0.013 | x |  |  |  |
| First 50 | 0.038 | 0.473 | x |  |  |
| Random 30 A | 0.063 | 0.527 | 1.000 | x |  |
| Random 30 B | 0.565 | 0.047 | 0.128 | 0.180 | x |
| All | 0.078 | 0.250 | 0.628 | 0.687 | 0.241 |

Supplementary Table S3(c). Multiple pairwise chi-square comparisons for the representation of dumbbell fibrils in different sub-samples for WH04.

| **LLM18** | Unaltered **n**/% | Beaded **n**/% | Dumbbell **n**/% | Total n |
| --- | --- | --- | --- | --- |
| First 30 | **0**/0% | **23**/79.3% | **6**/20.7% | **29** |
| Last 30 | **4**/13.8% | **21**/72.4% | **4**/13.8% | **29** |
| Random 30 A | **4**/13.3% | **20**/66.7% | **6**/20.0% | **30** |
| Random 30 B | **0**/0% | **23**/76.7% | **7**/23.3% | **30** |
| First 50 | **0**/0% | **39**/83.0% | **8**/17.0% | **47** |
| Original Counts (all) | **4**/6.9% | **44**/75.9% | **10**/17.2% | **58** |

Supplementary Table S4(a). Counts and percentages of classified fibrils by sub-sample of LLM18.


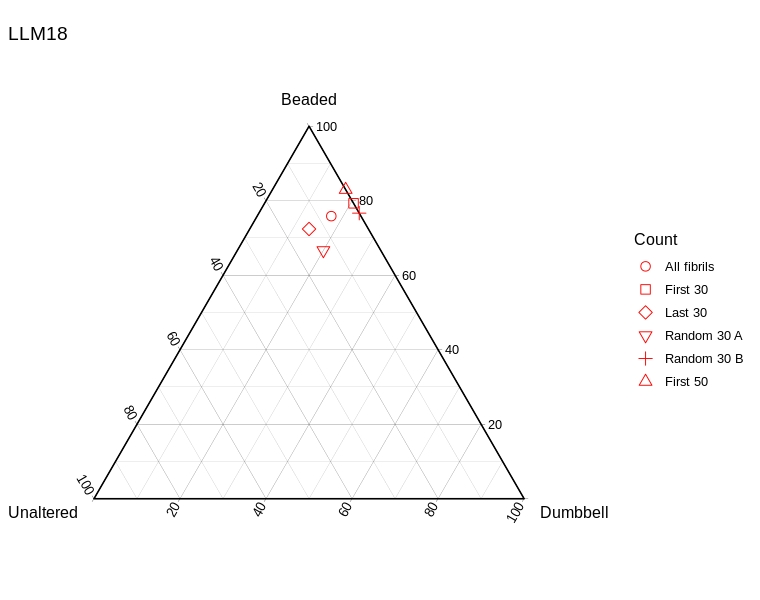


Supplementary Figure S4(b). Ternary plot representing the variation of re-assessed sub-samples of LLM18.

| **LLM18** (n=58) | 1st 30 | last 30 | first 50 | rand 30 1 | rand 30 2 |
| --- | --- | --- | --- | --- | --- |
| First 30 | x |  |  |  |  |
| Last 30 | 0.487 | x |  |  |  |
| First 50 | 0.689 | 0.708 | x |  |  |
| Random 30 A | 0.948 | 0.525 | 0.741 | x |  |
| Random 30 B | 0.807 | 0.347 | 0.495 | 0.754 | x |
| All | 0.696 | 0.680 | 0.976 | 0.750 | 0.493 |

Supplementary Table S4(c). Multiple pairwise chi-square comparisons for the representation of dumbbell fibrils in different sub-samples for LLM18.

| **PTN21** | Unaltered **n**/% | Beaded **n**/% | Dumbbell **n**/% | Total n |
| --- | --- | --- | --- | --- |
| First 30 | **0**/0% | **21**/63.6% | **12**/36.4% | **33** |
| Last 30 | **1**/3.1% | **23**/71.9% | **8**/25.0% | **32** |
| Random 30 A | **2**/6.7% | **21**/70.0% | **7**/23.3% | **30** |
| Random 30 B | **1**/3.3% | **20**/66.7% | **9**/30.0% | **30** |
| First 50 | **2**/3.8% | **35**/66.0% | **16**/30.2% | **53** |
| First 70 | **3**/4.3% | **49**/70.0% | **18**/25.7% | **70** |
| Original Counts (all) | **3**/3.9% | **53**/68.8% | **21**/27.3% | **77** |

Supplementary Table S5(a). Counts and percentages of classified fibrils by sub-sample of PTN21.


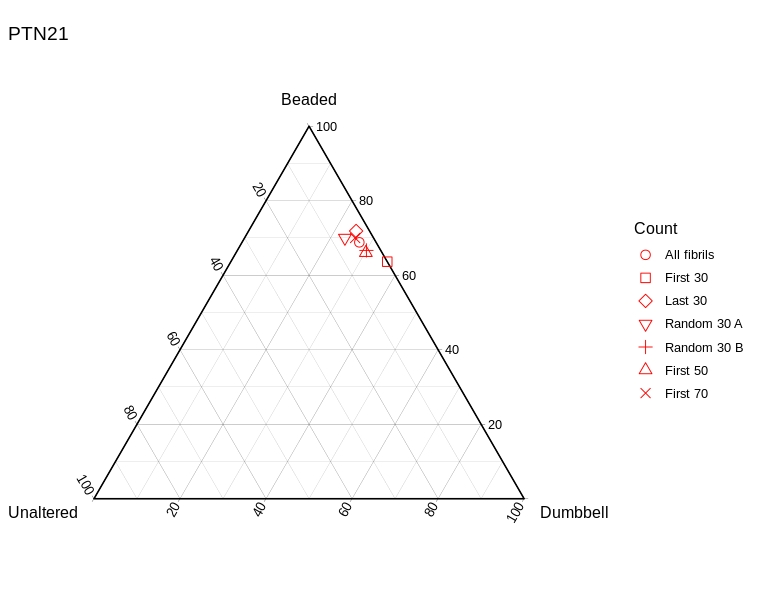


Supplementary Figure S5(b). Ternary plot representing the variation of re-assessed sub-samples of PTN21.

| **PTN21** (n=77) | First 30 | Last 30 | First 50 | Random 30 A | Random 30 B |
| --- | --- | --- | --- | --- | --- |
| First 30 | x |  |  |  |  |
| Last 30 | 0.321 | x |  |  |  |
| First 50 | 0.552 | 0.607 | x |  |  |
| Random 30 A | 0.260 | 0.878 | 0.503 | x |  |
| Random 30 B | 0.593 | 0.659 | 0.986 | 0.559 | x |
| All | 0.340 | 0.807 | 0.717 | 0.677 | 0.778 |

Supplementary Table S5(c). Multiple pairwise chi-square comparisons for the representation of dumbbell fibrils in different sub-samples for PTN21.

| **PTN22** | Unaltered **n**/% | Beaded **n**/% | Dumbbell **n**/% | Total n |
| --- | --- | --- | --- | --- |
| First 30 | **0**/0% | **22**/75.9% | **7**/24.1% | **29** |
| Last 30 | **0**/0% | **20**/69.0% | **9**/31.0% | **29** |
| Random 30 A | **0**/0% | **21**/70.0% | **9**/30.0% | **30** |
| Random 30 B | **0**/0% | 22/71.0% | **9**/29.0% | **31** |
| First 50 | **0**/0% | **37**/74.0% | **13**/26.0% | **50** |
| Original Counts (all) | **0**/0% | **41**/74.6% | **14**/25.5% | **55** |

Supplementary Table S6(a). Counts and percentages of classified fibrils by sub-sample of PTN22.


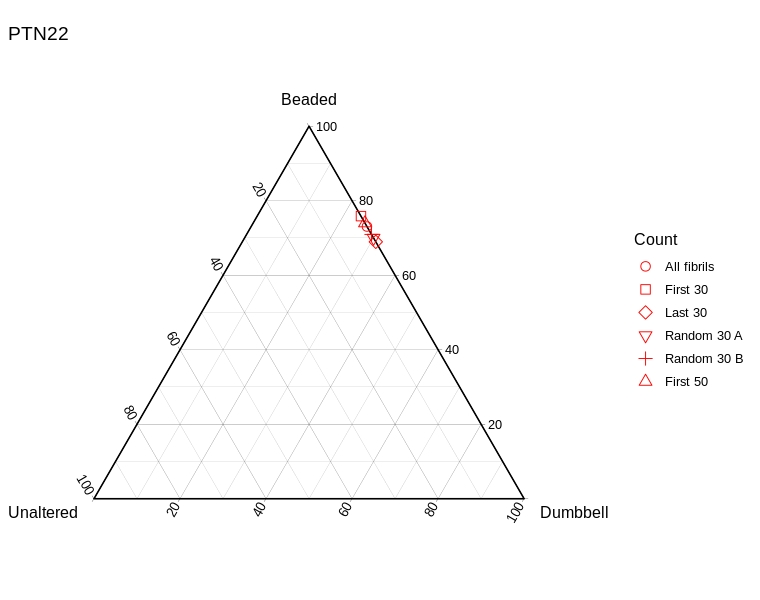


Supplementary Figure S6(b). Ternary plot representing the variation of re-assessed sub-samples of PTN22.

| **PTN22** (n=55) | First 30 | Last 30 | Random 30 A | Random 30 B |
| --- | --- | --- | --- | --- |
| First 30 | x |  |  |  |
| Last 30 | 0.557 | x |  |  |
| Random 30 A | 0.613 | 0.931 | x |  |
| Random 30 B | 0.668 | 0.866 | 0.934 | x |
| All | 0.895 | 0.586 | 0.652 | 0.719 |

Supplementary Table S6(c). Multiple pairwise chi-square comparisons for the representation of dumbbell fibrils in different sub-samples for PTN22.

| **PTN28** | Unaltered **n**/% | Beaded **n**/% | Dumbbell **n**/% | Total n |
| --- | --- | --- | --- | --- |
| First 30 | **0**/0% | **7**/23.3% | **23**/76.7% | **30** |
| Last 30 | **0**/0% | **9**/32.1% | **19**/67.9% | **28** |
| Random 30 A | **0**/0% | **12**/41.4% | **17**/58.6% | **29** |
| Random 30 B | **0**/0% | **12**/46.2% | **14**/53.8% | **26** |
| First 50 | **0**/0% | **16**/32.7% | **33**/67.4% | **49** |
| First 70 | **0**/0% | **24**/34.8% | **45**/65.2% | **69** |
| Original Counts (all) | **0**/0% | **25**/34.7% | **47**/65.3% | **72** |

Supplementary Table S7(a). Counts and percentages of classified fibrils by sub-sample of PTN28.


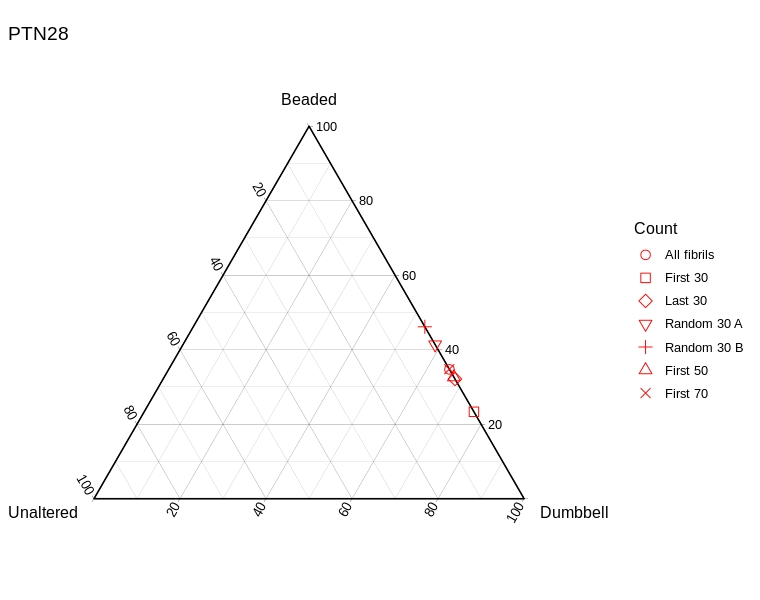


Supplementary Figure S7(b). Ternary plot representing the variation of re-assessed sub-samples of PTN28.

| **PTN28** (n=72) | First 30 | Last 30 | First 50 | Random 30 A | Random 30 B |
| --- | --- | --- | --- | --- | --- |
| First 30 | x |  |  |  |  |
| Last 30 | 0.453 | x |  |  |  |
| First 50 | 0.376 | 0.963 | x |  |  |
| Random 30 A | 0.138 | 0.470 | 0.437 | x |  |
| Random 30 B | 0.072 | 0.291 | 0.250 | 0.721 | x |
| All | 0.259 | 0.807 | 0.813 | 0.530 | 0.303 |

Supplementary Table S7(c). Multiple pairwise chi-square comparisons for the representation of dumbbell fibrils in different sub-samples for PTN28.

| **ECH31** | Unaltered **n**/% | Beaded **n**/% | Dumbbell **n**/% | Total n |
| --- | --- | --- | --- | --- |
| First 30 | **0**/0% | **20**/60.6% | **13**/39.4% | **33** |
| Last 30 | **0**/0% | **4**/13.8% | **25**/86.2% | **29** |
| Random 30 A | **0**/0% | **16**/47.1% | **18**/52.9% | **34** |
| Random 30 B | **0**/0% | **16**/50.0% | **16**/50.0% | **32** |
| Original Counts (all) | **0**/0% | **20**/38.5% | **32**/61.5% | **52** |

Supplementary Table S8(a). Counts and percentages of classified fibrils by sub-sample of ECH31.


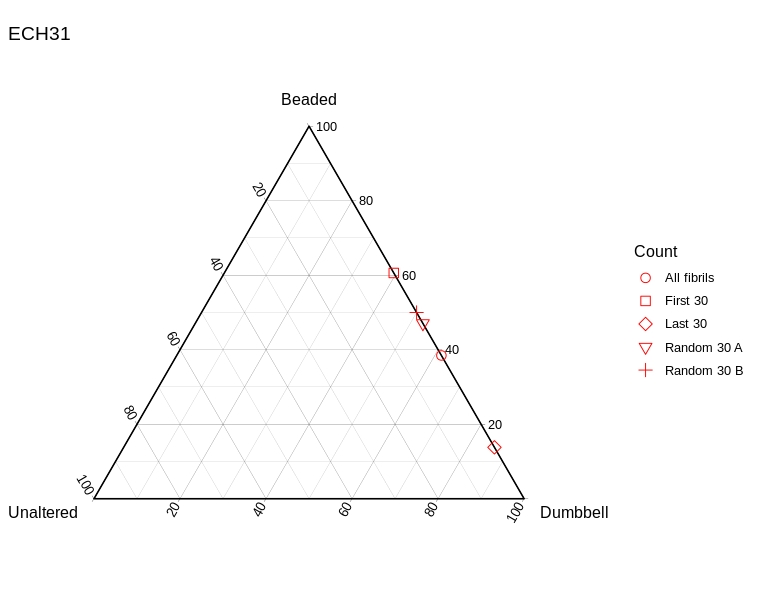


Supplementary Figure S8(b). Ternary plot representing the variation of re-assessed sub-samples of ECH31.

| **ECH31** (n=52) | First 30 | Last 30 | Random 30 A | Random 30 B |
| --- | --- | --- | --- | --- |
| First 30 | x |  |  |  |
| Last 30 | <0.001 | x |  |  |
| Random 30 A | 0.266 | 0.003 | x |  |
| Random 30 B | 0.390 | 0.005 | 0.811 | x |
| All | 0.046 | 0.020 | 0.429 | 0.299 |

Supplementary Table S8(c). Multiple pairwise chi-square comparisons for the representation of dumbbell fibrils in different sub-samples for ECH31.

| **ECH33** | Unaltered **n**/% | Beaded **n**/% | Dumbbell **n**/% | Total n |
| --- | --- | --- | --- | --- |
| First 30 | **2**/6.7% | **20**/66.7% | **8**/26.7% | **30** |
| Last 30 | **0**/0% | **16**/51.6% | **15**/48.4% | **31** |
| Random 30 A | **0**/0% | **14**/45.2% | **17**/54.8% | **31** |
| Random 30 B | **2**/6.7% | **15**/50.0% | **13**/43.3% | **30** |
| First 50 | **2**/4.0% | **32**/64.0% | **16**/32.0% | **50** |
| Original Counts (all) | **2**/2.8% | **40**/56.3% | **29**/40.9% | **71** |

Supplementary Table S9(a). Counts and percentages of classified fibrils by sub-sample of ECH33.


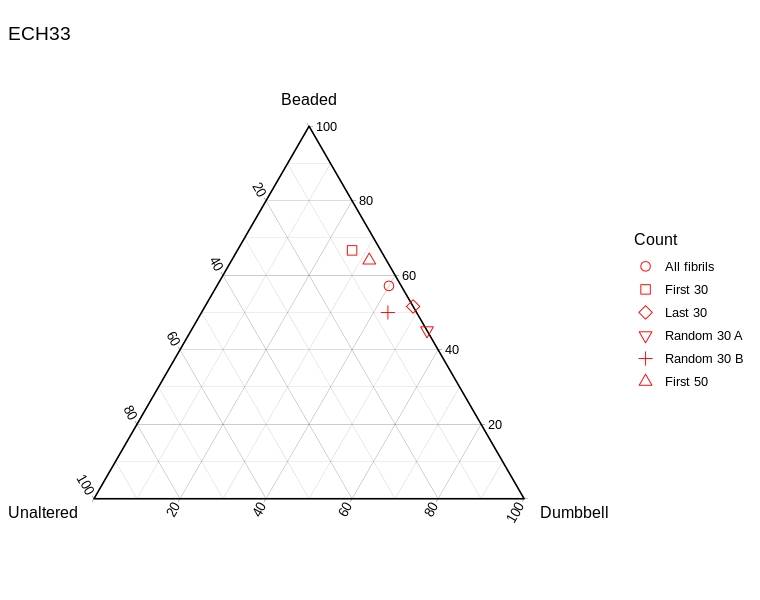


Supplementary Figure S9(b). Ternary plot representing the variation of re-assessed sub-samples of ECH33.

| **ECH33** (n=71) | First 30 | Last 30 | First 50 | Random 30 A | Random 30 B |
| --- | --- | --- | --- | --- | --- |
| First 30 | x |  |  |  |  |
| Last 30 | 0.080 | x |  |  |  |
| First 50 | 0.614 | 0.140 | x |  |  |
| Random 30 A | 0.025 | 0.611 | 0.042 | x |  |
| Random 30 B | 0.176 | 0.692 | 0.307 | 0.369 | x |
| All | 0.177 | 0.479 | 0.322 | 0.191 | 0.817 |

Supplementary Table S9(c). Multiple pairwise chi-square comparisons for the representation of dumbbell fibrils in different sub-samples for ECH33.

| **ECH35** | Unaltered **n**/% | Beaded **n**/% | Dumbbell **n**/% | Total n |
| --- | --- | --- | --- | --- |
| First 30 | **0**/0.0% | **21**/72.4% | **8**/27.6% | **29** |
| Last 30 | **3**/10.3% | **18**/62.1% | **8**/27.6% | **29** |
| Random 30 A | **1**/3.5% | **20**/69.0% | **8**/27.6% | **29** |
| Random 30 B | **3**/9.7% | **22**/71.0% | **6**/19.4% | **31** |
| First 50 | **3**/6.3% | **32**/66.7% | **13**/27.1% | **48** |
| Original Counts (all) | **3**/5.7% | **34**/64.2% | **16**/30.2% | **53** |

Supplementary Table S10(a). Counts and percentages of classified fibrils by sub-sample of ECH35.


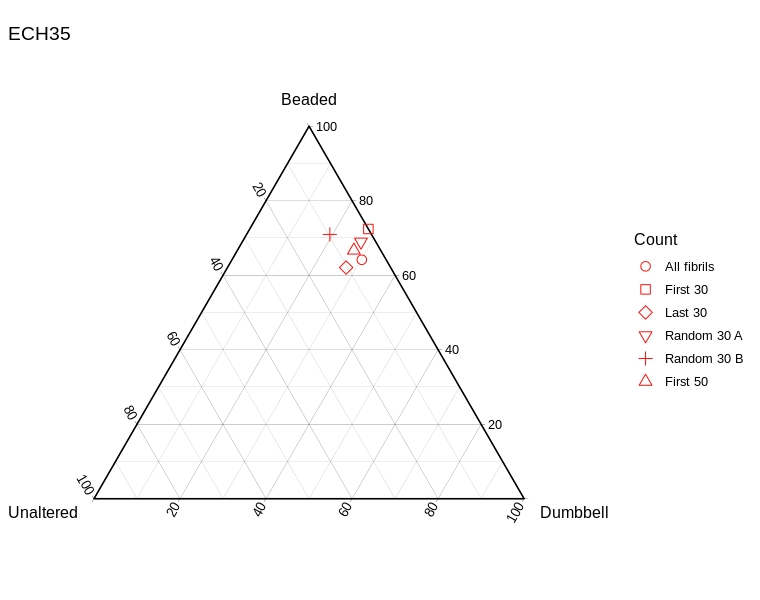


Supplementary Figure S10(b). Ternary plot representing the variation of re-assessed sub-samples of ECH35.

| **ECH35** (n=53) | First 30 | Last 30 | Random 30 A | Random 30 B |
| --- | --- | --- | --- | --- |
| First 30 | x |  |  |  |
| Last 30 | 1.000 | x |  |  |
| Random 30 A | 1.000 | 1.000 | x |  |
| Random 30 B | 0.451 | 0.451 | 0.451 | x |
| All | 0.804 | 0.804 | 0.804 | 0.276 |

Supplementary Table S10(c). Multiple pairwise chi-square comparisons for the representation of dumbbell fibrils in different sub-samples for ECH35.

| **ECH36** | Unaltered **n**/% | Beaded **n**/% | Dumbbell **n**/% | Total n |
| --- | --- | --- | --- | --- |
| First 30 | **5**/17.2% | **19**/65.5% | **5**/17.2% | **29** |
| Last 30 | **4**/12.9% | **23**/74.2% | **4**/12.9% | **31** |
| Random 30 A | **4**/14.3% | **23**/82.1% | **1**/3.6% | **28** |
| Random 30 B | **3**/10.0% | **22**/73.3% | **5**/16.7% | **30** |
| First 50 | **5**/10.0% | **33**/66.0% | **12**/24.0% | **50** |
| First 70 | **9**/12.7% | **48**/67.6% | **14**/19.7% | **71** |
| Original Counts (all) | **9**/11.0% | **57**/69.5% | **16**/19.5% | **82** |

Supplementary Table S11(a). Counts and percentages of classified fibrils by sub-sample of ECH36.


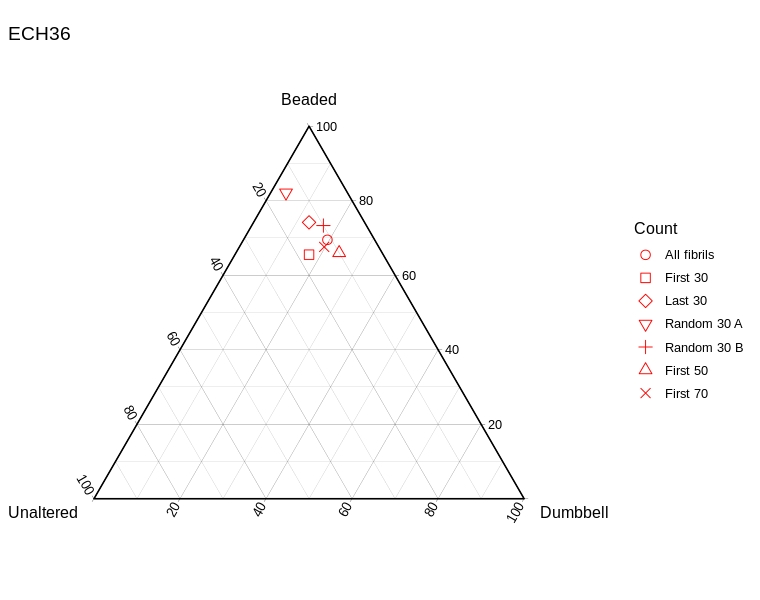


Supplementary Figure S11(b). Ternary plot representing the variation of re-assessed sub-samples of ECH36.

| **ECH36** (n=82) | First 30 | Last 30 | First 50 | First 70 | Random 30 A | Random 30 B |
| --- | --- | --- | --- | --- | --- | --- |
| First 30 | x |  |  |  |  |  |
| Last 30 | 0.638 | x |  |  |  |  |
| First 50 | 0.481 | 0.223 | x |  |  |  |
| First 70 | 0.774 | 0.406 | 0.572 | x |  |  |
| Random 30 A | 0.093 | 0.199 | 0.020 | 0.044 | x |  |
| Random 30 B | 0.953 | 0.679 | 0.438 | 0.720 | 0.102 | x |
| All | 0.788 | 0.411 | 0.541 | 0.974 | 0.044 | 0.733 |

Supplementary Table S11(c). Multiple pairwise chi-square comparisons for the representation of dumbbell fibrils in different sub-samples for ECH36.

| **ECH37** | Unaltered **n**/% | Beaded **n**/% | Dumbbell **n**/% | Total n |
| --- | --- | --- | --- | --- |
| First 30 | **1**/3.5% | **14**/48.3% | **14**/48.3% | **29** |
| Last 30 | **2**/6.5% | **16**/51.6% | **13**/41.9% | **31** |
| Random 30 A | **2**/6.5% | **11**/35.5% | **18**/58.1% | **31** |
| Random 30 B | **1**/3.5% | **14**/48.3% | **14**/48.3% | **29** |
| First 50 | **3**/6.0% | **25**/50.0% | **22**/44.0% | **50** |
| Original Counts (all) | **3**/5.0% | **30**/50.0% | **27**/45.0% | **60** |

Supplementary Table S12(a). Counts and percentages of classified fibrils by sub-sample of ECH37.


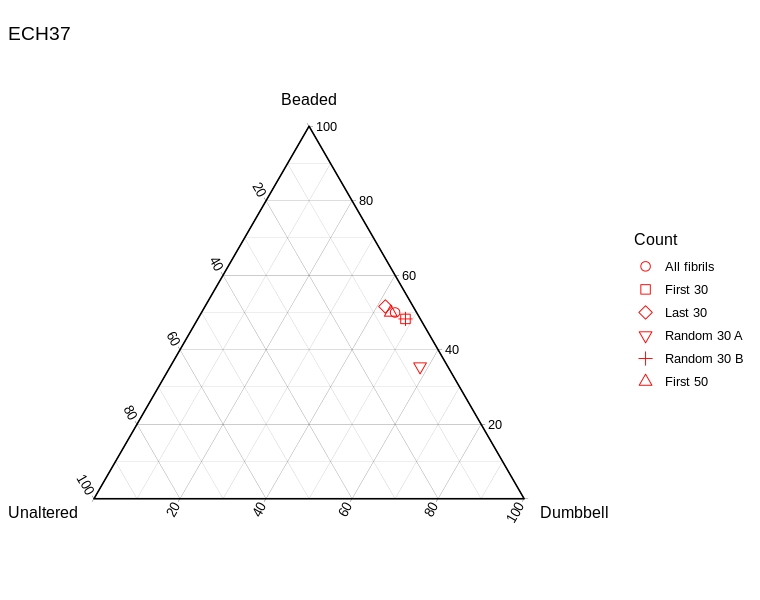


Supplementary Figure S12(b). Ternary plot representing the variation of re-assessed sub-samples of ECH37.

| **ECH37** (n=60) | First 30 | Last 30 | First 50 | Random 30 A | Random 30 B |
| --- | --- | --- | --- | --- | --- |
| First 30 | x |  |  |  |  |
| Last 30 | 0.622 | x |  |  |  |
| First 50 | 0.713 | 0.855 | x |  |  |
| Random 30 A | 0.448 | 0.204 | 0.218 | x |  |
| Random 30 B | 1.000 | 0.622 | 0.713 | 0.448 | x |
| All | 0.771 | 0.780 | 0.916 | 0.237 | 0.771 |

Supplementary Table S12(c). Multiple pairwise chi-square comparisons for the representation of dumbbell fibrils in different sub-samples for ECH37.

| **ECH39** | Unaltered **n**/% | Beaded **n**/% | Dumbbell **n**/% | Total n |
| --- | --- | --- | --- | --- |
| First 30 | **1**/3.5% | **19**/65.5% | **9**/31.0% | **29** |
| Last 30 | **3**/9.4% | **21**/65.6% | **8**/25.0% | **32** |
| Random 30 A | **2**/6.3% | **21**/65.6% | **9**/28.1% | **32** |
| Random 30 B | **2**/6.7% | **21**/70.0% | **7**/23.3% | **30** |
| First 50 | **4**/7.7% | **34**/65.4% | **14**/26.9% | **52** |
| Original Counts (all) | **4**/5.7% | **47**/67.1% | **19**/27.1% | **70** |

Supplementary Table S13(a). Counts and percentages of classified fibrils by sub-sample of ECH39.


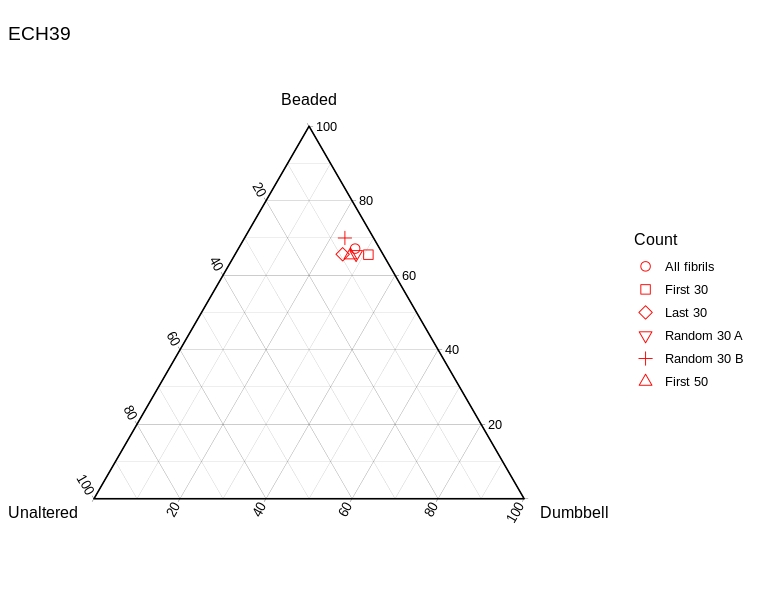


Supplementary Figure S13(b). Ternary plot representing the variation of re-assessed sub-samples of ECH39.

| **ECH39** (n=70) | First 30 | Last 30 | First 50 | Random 30 A | Random 30 B |
| --- | --- | --- | --- | --- | --- |
| First30 | x |  |  |  |  |
| Last 30 | 0.600 | x |  |  |  |
| First 50 | 0.694 | 0.846 | x |  |  |
| Random 30 A | 0.804 | 0.777 | 0.905 | x |  |
| Random 30 B | 0.506 | 0.878 | 0.720 | 0.667 | x |
| All | 0.696 | 0.820 | 0.978 | 0.918 | 0.691 |

Supplementary Table S13(c). Multiple pairwise chi-square comparisons for the representation of dumbbell fibrils in different sub-samples for ECH39.

| **ECH40** | Unaltered **n**/% | Beaded **n**/% | Dumbbell **n**/% | Total n |
| --- | --- | --- | --- | --- |
| First 30 | **6**/18.8% | **21**/65.6% | **5**/15.6% | **32** |
| Last 30 | **4**/12.9% | **20**/64.5% | **7**/22.6% | **31** |
| Random 30 A | **6**/20.7% | **18**/62.1% | **5**/17.2% | **29** |
| Random 30 B | **7**/21.9% | **22**/68.8% | **3**/9.4% | **32** |
| First 50 | **10**/19.6% | **34**/66.7% | **7**/13.7% | **51** |
| Original Counts (all) | **13**/18.3% | **46**/64.8% | **12**/16.9% | **71** |

Supplementary Table S14(a). Counts and percentages of classified fibrils by sub-sample of ECH40.


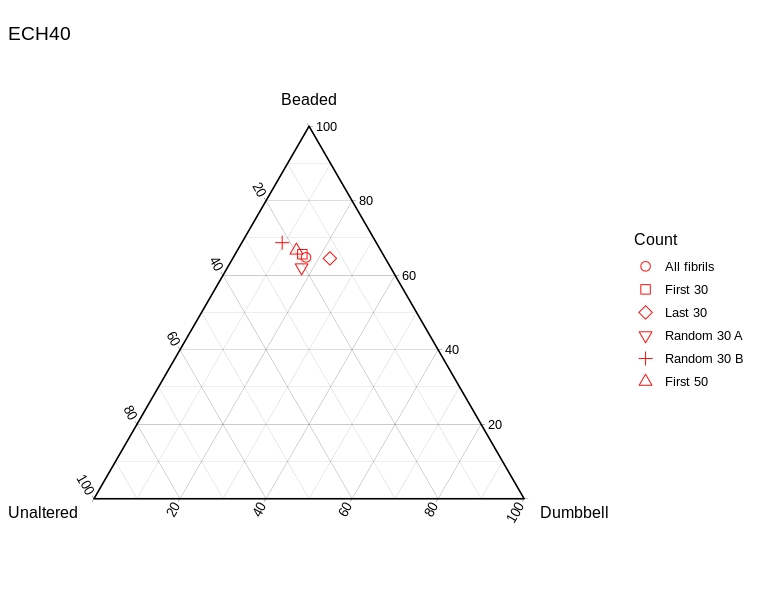


Supplementary Figure S14(b). Ternary plot representing the variation of re-assessed subsamples of ECH40.

| **ECH40** (n=71) | First 30 | Last 30 | First 50 | Random 30 A | Random 30 B |
| --- | --- | --- | --- | --- | --- |
| First30 | x |  |  |  |  |
| Last 30 | 0.482 | x |  |  |  |
| First 50 | 0.811 | 0.301 | x |  |  |
| Random 30 A | 0.865 | 0.605 | 0.672 | x |  |
| Random 30 B | 0.450 | 0.152 | 0.553 | 0.363 | x |
| All | 0.872 | 0.498 | 0.633 | 0.967 | 0.316 |

Supplementary Table S146(c). Multiple pairwise chi-square comparisons for the representation of dumbbell fibrils in different sub-samples for ECH40.
